# Evolutionary Analysis of Cnidaria Small Cysteine-Rich Proteins (SCRiPs), an Enigmatic Neurotoxin Family from Stony Corals and Sea Anemones (Anthozoa: Hexacorallia)

**DOI:** 10.3390/toxins16020075

**Published:** 2024-02-02

**Authors:** Ricardo Alexandre Barroso, Luana Ramos, Hugo Moreno, Agostinho Antunes

**Affiliations:** 1 CIIMAR/CIMAR, Interdisciplinary Centre of Marine and Environmental Research, University of Porto, Terminal de Cruzeiros do Porto de Leixões, Av. General Norton de Matos, s/n, 4450-208 Porto, Portugal; barrosoalex98@gmail.com (R.A.B.); luana.fscramos@gmail.com (L.R.); hugocosmoreno@gmail.com (H.M.); 2 Department of Biology, Faculty of Sciences, University of Porto, Rua do Campo Alegre, 4169-007 Porto, Portugal

**Keywords:** cnidaria, SCRiP, toxin, evolution

## Abstract

Cnidarians (corals, sea anemones, and jellyfish) produce toxins that play central roles in key ecological processes, including predation, defense, and competition, being the oldest extant venomous animal lineage. Cnidaria small cysteine-rich proteins (SCRiPs) were the first family of neurotoxins detected in stony corals, one of the ocean’s most crucial foundation species. Yet, their molecular evolution remains poorly understood. Moreover, the lack of a clear classification system has hindered the establishment of an accurate and phylogenetically informed nomenclature. In this study, we extensively surveyed 117 genomes and 103 transcriptomes of cnidarians to identify orthologous *SCRiP* gene sequences. We annotated a total of 168 novel putative *SCRiPs* from over 36 species of stony corals and 12 species of sea anemones. Phylogenetic reconstruction identified four distinct *SCRiP* subfamilies, according to strict discrimination criteria based on well-supported monophyly with a high percentage of nucleotide and amino acids’ identity. Although there is a high prevalence of purifying selection for most *SCRiP* subfamilies, with few positively selected sites detected, a subset of Acroporidae sequences is influenced by diversifying positive selection, suggesting potential neofunctionalizations related to the fine-tuning of toxin potency. We propose a new nomenclature classification system relying on the phylogenetic distribution and evolution of *SCRiPs* across Anthozoa, which will further assist future proteomic and functional research efforts.

## 1. Introduction

Cnidarians are the oldest extant lineage of venomous animals [1,2,3,4], with more than 11,000 described species [5]. This phylum emerged during the Cryogenian-to-Tonian period, around 798 Mya [6,7,8], being the sister-group of Bilateria in metazoans [9]. Cnidaria is established by three major clades—the sessile Anthozoa, divided in the subclasses Hexacorallia (e.g., stony corals and sea anemones), Ceriantharia (tube-dwelling anemones/ceriantharians), and Octocorallia (e.g., soft corals, sea pans and sea fans); the free-living Medusozoa (e.g., jellyfish and hydrozoans); and the microscopical endoparasites Myxozoa [8,9,10,11,12]. Cnidarians subdue prey and protect themselves through the injection of stinging structures filled with venom—nematocysts—into the target organisms [1,13,14], and their venomous arsenal varies between groups [15,16,17,18,19,20]. Currently, research efforts are mostly focused on cnidaria venom composition and pharmacological effects [21,22,23], neglecting their ecological relevance. Only more recently, multi-omics tools contributed to the expansion of evolutionary studies in cnidaria toxins [24,25,26,27,28,29], including in stony corals (Scleractinia) [30,31], whose venomous traits are largely unexplored [32].

The first major toxin family detected in stony corals was the cnidaria small cysteine-rich proteins (SCRiP). SCRiPs were first discovered in silico by an initial search for antimicrobial peptides (AMPs), particularly β-defensin-like AMPs, in expressed sequence tag (EST) libraries from the stony corals *Orbicella faveolata* (prior *Montastraea faveolata*), *Montipora capitata*, and *Acropora millepora* [33]. Like other toxins, SCRiPs are secretory [34,35], containing a hydrophobic signal peptide. Some exhibit potential proprotein convertase (PC) cleavage sites, with the preprotein being around 68–83 amino acids (aa) in length, the proprotein being 53–62 aa, and the mature protein being 40–48 aa. The molecular weight of mature proteins ranges from 4.3 to 5.8 KDa [33]. Their most remarkable characteristic is the conserved eight-cysteine framework with three cysteines together in the C-terminal region [28,33]. 

SCRiPs were previously thought to be involved in calcifying roles, as they were identified as unique genes in stony corals and exhibited a similar downregulated temporal expression pattern to that of galaxin, a key protein involved in the biomineralization process, during heat stress [33]. However, SCRiPs were also detected in sea anemones [24,28,36], which do not calcify, refuting this statement. Moreover, zebrafish (*Danio rerio*) larvae incubated with two recombinantly expressed SCRiPs from *A. millepora* at a concentration of 230 µg/mL exhibited abnormal neurotoxic signals, such as twitching and shivering, followed by paralysis and death, suggesting that SCRiPs are neurotoxins [28]. From these, Amil_SCRiP2 (UniProt: COH691) was more potent than Amil_SCRiP3 (UniProt: COH692) (death after 200 min and 16 h, respectively) [28]. Later, a cDNA sequence member of the SCRiP family was found in the ectoderm of *A. millepora* via in situ hybridization, which is filled with nematocytes [37], and one of the most expressed toxin genes in the branches of the stony coral *Stylophora pistillata* was a *SCRiP* [38], reinforcing this assumption. *SCRiPs* are also highly duplicated in stony coral genomes, which could contribute to their ecological success [39], and some may be differentially expressed in distinct life stages of *A. millepora* [33] and *Acropora digitifera* [39]. In fact, gene duplication has a substantial role in shaping the venom repertoire of anthozoans [31,36,40].

**Figure 1:**
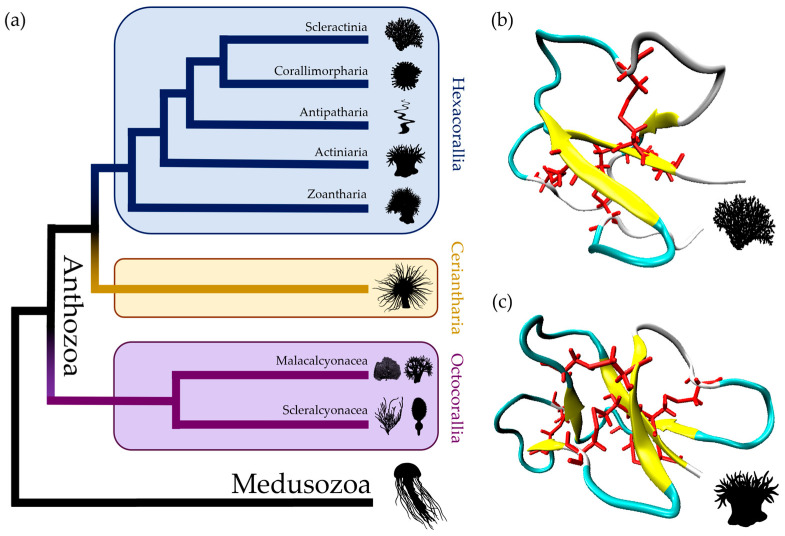
(**a**) Simplified phylogenetic tree of Anthozoa [8,11,12], considering recent taxonomic revisions [10,43]. Each sub-class from Anthozoa is colored differently. SCRiPs detected at the protein level in Anthozoa are represented by the 3D structures of (**b**) Hact_SCRiP1 (PDB: 7LX4) from the stony coral *Heliofungia actiniformis*, with 4 disulfide bonds; and the SCRiP-like peptide (**c**) Ueq 12-1 (PDB: 5LAH) from the sea anemone *Urticina eques*, with 5 disulfide bonds. Cysteines are represented in red. Protein structures were rendered in VMD: https://www.ks.uiuc.edu/Research/vmd/ (accessed on 16 September 2023).

Recently, SCRiPs were isolated at the protein level in nematocyst extracts from the tentacles of the stony coral *Heliofungia actiniformis* (Hact_SCRiP1; PDB: 7LX4) [41] (Figure 1b), and a remarkable SCRiP-like peptide with a similar cysteine framework from ectoderm secretions of the sea anemone *Urticina eques* (Ueq 12-1; PDB: 5LAH) was also isolated [42] (Figure 1c), suggesting that SCRiPs’ transcripts are true toxin-coding genes. Both peptides have a β-defensin-like fold, with Ueq 12-1 showing antimicrobial and transient receptor potential ankyrin 1 (TRPA1) potentiating properties (analgesic) [42]. The dual effect of Ueq 12-1 may explain the function of active predation by neurotoxin production, while resisting bacterial infections caused by eventual tentacle injury [42]. 

The lack of information about the evolution and phylogenetic distribution of SCRiPs in cnidarians may cause misunderstanding for potential research on this family of toxins. Moreover, SCRiPs lack a well-supported nomenclature system, as they were not named based on sequence similarity, but on the order in which they were discovered. Here, we intend to fill these gaps for future proteomic and functional studies.

## 2. Results

### 2.1. SCRiPs Have a Wide Distribution within Stony Corals (Scleractinia) and Sea Anemones (Actiniaria)

**Figure 2:**
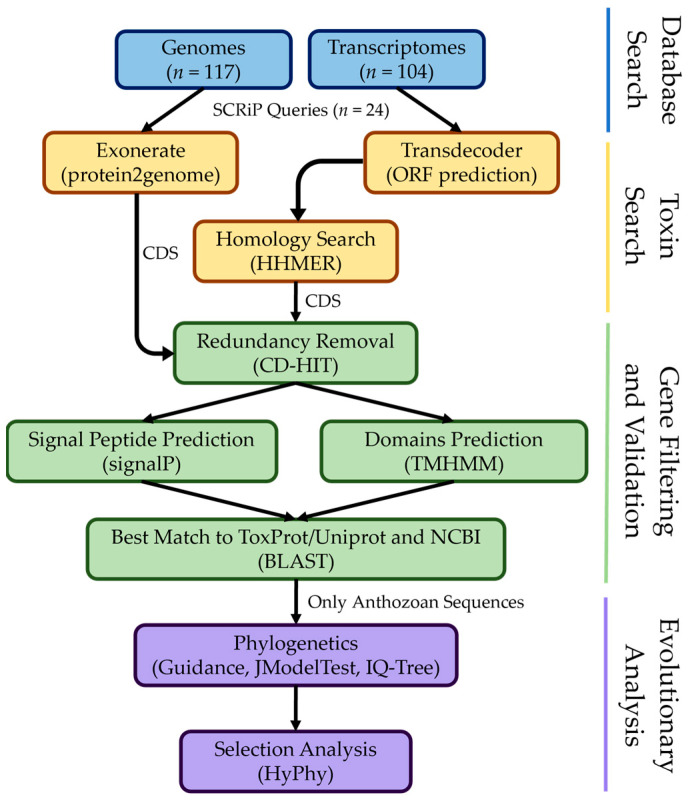
Bioinformatic pipeline applied in this study for the identification, filtering, validation, and evolutionary analyses of *SCRiPs* extracted from genomic and transcriptomic data of Cnidaria. CDS—complete coding sequence.

From an initial search in 117 genomes and 104 transcriptome assemblies from Cnidaria (Supplementary Table S1), using 24 SCRiP queries, we identified a total of 426 potential *SCRiP* sequences. However, after filtering and gene validation (see bioinformatic pipeline, Figure 2), the final dataset consisted of 168 new putative *SCRiP* sequences, half of those from genomic data (84) and the other half from transcriptomic data (Supplementary Table S2). These sequences belong to 36 species of stony corals and 12 species of sea anemones, both from Hexacorallia (Figure 1). A total of 106 (63.09%) sequences obtained from the genomes and transcriptomes are complete, while 62 (36.91%) are partial sequences (smaller sequences with high similarity with the queries) obtained only from the genomes. These were selected for phylogenetic analysis, along with the 24 query sequences (Table 1), compiling 192 *SCRiP* sequences: 173 (90.10%) from 37 stony corals and 19 (9.90%) from 14 sea anemones. We found *SCRiP* sequences with 100% similarity within *Acropora* spp., as well as in the transcriptomes of the sea anemones *Entacmaea quadricolor* and *Heteractis aurora* (Supplementary Table S2).

No *SCRiP* sequences were identified in Corallimorpharia, Zoantharia (Hexacorallia), and Ceriantharia data, nor in Scleralcyonacea and Malacalcyonacea (Octocorallia) data, except for the presence of five *SCRiP*-like sequences in the malacalcyonacean *Scleronephthya gracillima*. Curiously, we also identified two putative *SCRiPs* from two species of Cubozoa that passed our filtering criteria: *Copula sivickisi* and *Morbakka virulenta* (Supplementary Table S2), which belong within Medusozoa and, therefore, were not considered for further analysis. *SCRiPs* were not detected in the non-venomous phylum Ctenophora, but four *SCRiPs* were detected in two (12.5%) *Symbiodinium* spp. transcriptomes. A *SCRiP* sequence from the endosymbiotic algae *Symbiodinium muscatinei* (GenBank: GFDR00000000.3), with the sea anemone *Anthopleura elegantissima* as host, has a 96.67% identity to the *SCRiP* retrieved from the *A. elegantissima* transcriptome in this study (Supplementary File S1).

### 2.2. A New SCRiP Nomenclature Supported by Phylogenetic Data

**Figure 3:**
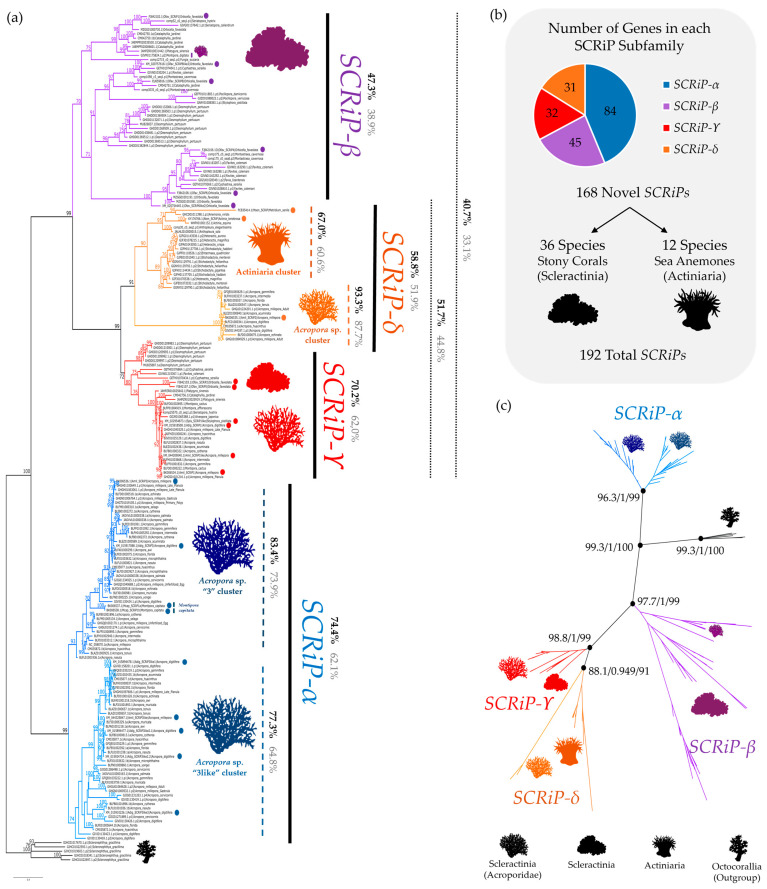
(**a**) Maximum Likelihood (ML) gene tree of *SCRiP* nucleotide sequences. Phylogeny was constructed using IQ-Tree and 10,000 ultrafast bootstraps replicates. Bootstrap values above 70% are shown above the branches. Nucleotide identity (ni) and protein identity (pi) (%) are represented in each *SCRiP* subfamily in black and gray, respectively. Colored dots mark *SCRiP* sequences previously identified in other studies and available in public databases. (**b**) Number of *SCRiPs* identified in each subfamily. (**c**) The radiation tree of *SCRiPs* includes statistics for the parametric approximate likelihood-ratio test (SH-aLRT), approximate Bayes test, and ultrafast bootstraps in the divergence nodes, respectively. See Supplementary Figure S1 for the remaining values.

Considering the *SCRiP*-like sequences from the octocoral *S. gracillima* as an outgroup, the Maximum Likelihood (ML) nucleotide gene tree formed four monophyletic clades with strong bootstrap support (bs) values (Figure 3a,b). Moreover, the parametric approximate likelihood-ratio test (SH-aLRT) and approximate Bayes test also support the division of the *SCRiP* subfamilies in the ML gene tree (Figure 3c and Supplementary Figure S1). The nomenclature of the SCRiP family was given in alphabetic order, starting with the first major clade in the ML tree.

The *SCRiP-α* (*alfa*) clade (bs = 99%; nucleotide identity (ni) = 74.4%) seems to be further divided into two subgroups, one containing previous SCRiP3 and Mcap_SCRiP queries (“*Acropora* sp. 3 cluster”; bs = 93%; ni = 83.4%) and another with previous SCRiP3like queries (“*Acropora* sp. 3like cluster”; bs = 74%; ni = 77.3%). However, given the high-percentage identity (ni = 74.4%) of the *SCRiP-α* group, we considered it one major group. *SCRiP-α* is formed by 84 sequences of stony corals of the family Acroporidae (Table 2), with 2 (2.4%) belonging to *M. capitata* and 82 (97.6%) to 18 species of the *Acropora* genus.

Our phylogenetic results show that the *SCRiP-α* sister clade (bs = 99%) is further divided into the *SCRiP-β* (*beta*) clade (bs = 70%; ni = 47.3%), and another (bs = 99%) constituted by *SCRiP-ϒ* (*gamma*) (bs = 72%; ni = 70.2%) and *SCRiP-δ* (*delta*) (bs = 91%; ni = 58.8%).

*SCRiP-β* subfamily encompasses the highest taxonomic diversity, formed by 45 sequences from over 15 species of 7 families of stony corals, including 1 (2.22%) from Acroporidae, 10 (22.22%) from Caryophylliidae, 1 (2.22%) from Faviidae, 1 (2.22%) from Fungiidae, 23 (51.11%) from Merulinidae, 4 (8.89%) from Montastraeidae, and 5 (11.11%) from Pocilloporidae (Table 2). Interestingly, *SCRiP-β* does not contain any *Acropora* sp. sequences.

*SCRiP-ϒ* is formed by 32 sequences from over 18 species of 4 families of stony corals, including 16 (50%) from Acroporidae, 6 (18.75%) from Caryophylliidae, 8 (25.00%) from Merulinidae, and 2 (6.25%) from Pocilloporidae (Table 2).

*SCRiP-δ* is formed by 31 sequences, including 19 (61.3%) from sea anemones that cluster together (“Actiniaria cluster”; bs = 90%; ni = 67.00%) and 12 (38.70%) from stony corals of the genus *Acropora* that also cluster together (“*Acropora* sp. cluster”; bs = 100%; ni = 93.30). Within the 19 sea anemone sequences, 18 (94.74%) belong to the Actinioidea superfamily (Actiniidae, Heteractidae, and Stichodactylidae) and 1 (5.26%) to the Metridioidea superfamily (Metridiidae) (Table 2).

**Figure 4:**
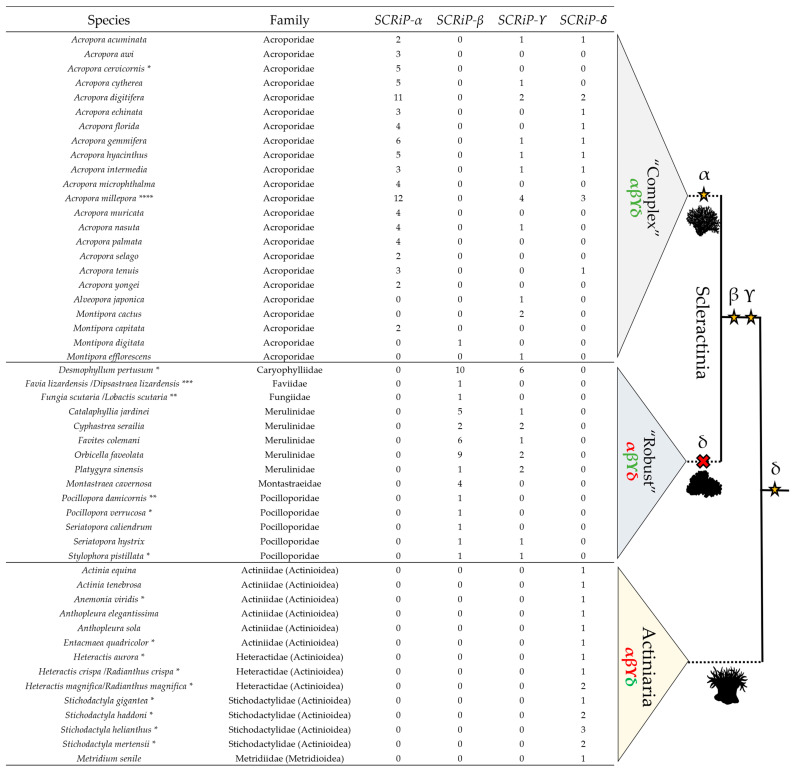
Number of *SCRiP* orthologs (*n* = 192) from each *SCRiP* subfamily expressed in stony corals (Scleractinia) and sea anemones (Actiniaria). Phylogeny of “complex” and “robust” stony corals is adapted from previous phylogenetic studies of Scleractinia [44,45]. *SCRiPs* from each phylogenetic clade are colored according to their expression: green means that there is evidence of expression, and red means that it was not detected in any species. Stars and crosses indicate the birth and death of a gene, respectively. Transcriptomes with available sample information are marked with asterisks: * adults, ** larvae, *** gastrula, and **** all life stages. In Actiniaria families, the superfamily is indicated in brackets, and the *SCRiPs* identified in adults belong to the tentacles (except for *Anemonia viridis*, which is unknown). Species names were verified using the WoRMS database [43].

*SCRiP* subfamilies are distributed among phylogenetically distinct groups within Actiniaria and Scleractinia (Figure 3), suggesting potential gene losses and gains throughout evolution. Sea anemones only express *SCRiP-δ*. Within stony corals, “complex” stony corals express all *SCRiP* subfamilies, while “robust” stony corals only express *SCRiP-β* and *SCRiP-ϒ* (Figure 4).

### 2.3. Selection Analysis Reveal Different Evolutionary Constrains

The *SCRiP-β*, *SCRiP-ϒ*, and *SCRiP-δ* subfamilies are under the broad influence of purifying selection (*ω* < 1) (mean of 0.638, 0.619, and 0.576, respectively), while *SCRiP-α* is under diversifying positive selection (*ω* > 1), as shown by the global *ω* values from the MG94xREV model (mean of 1.30) and the presence of more positively selected sites (PSSs) (Table 3). Nevertheless, all clades show signals of PSSs across tests (Supplementary Table S3).

### 2.4. SCRiPs May Be Differentially Expressed across Stony Corals and Sea Anemones

*SCRiP* subfamilies were detected in transcriptomes of stony corals belonging to distinct life stages: *Acropora cervicornis* (*SCRiP-α)*, *Desmophyllum pertusum* (*SCRiP-β* and *SCRiP-ϒ*), *Pocillopora verrucosa* (*SCRiP-β*), and *S. pistillata* (*SCRiP-β* and *SCRiP-ϒ*) from adults; *Fungia scutaria* (*SCRiP-β*) and *Pocillopora damicornis* (*SCRiP-β*) from larvae; and *Favia lizardensis* (*SCRiP-β*) from gastrula (Figure 4 and Supplementary Table S2). In the case of *A. millepora*, *SCRiP-α* paralogs are present throughout the entire life cycle. However, *SCRiP-ϒ* is not expressed in the gastrula, post-gastrula, and adult phases, while *SCRiP-δ* was not detected in the unfertilized egg (Supplementary Table S2).

Conversely, in sea anemones from the Actinioidea superfamily (13), more than half of the *SCRiP-δ* orthologs were known to be retrieved from adults (69.2%). Of these nine adults, eight samples belonged to the tentacles (88.89%) (Figure 4 and Supplementary Table S2).

## 3. Discussion

### 3.1. SCRiPs Are Common in Several Families of Stony Corals (Scleractinia) and Sea Anemones (Actiniaria)

Stony corals are considerably less explored regarding their venom content compared with sea anemones [18], being an interesting target for ecological, evolutionary, and bioprospecting studies of their toxins. Curiously, these two orders express the same family of neurotoxins—SCRiPs [24,28,36,41]—constituting the first characterized toxins from stony corals [32]. Here, we detected 168 new *SCRiP* sequences from 36 species of stony corals and 12 species of sea anemones following a strict bioinformatic pipeline (Figure 2), a major improvement from the 14 SCRiP sequences currently reviewed in *Uniprot*.

*SCRiPs* were detected in seven families of stony corals—Acroporidae, Caryophylliidae, Faviidae, Fungiidae, Merulinidae, Montastraeidae, and Pocilloporidae—demonstrating their higher phylogenetic distribution. A SCRiP peptide was already isolated from the nematocysts of a species from the family Fungiidae—*Heliofungia actiniformis* [41]. Moreover, a previous study demonstrated the presence of unknown peptides with toxic activity and low molecular weights in nematocyst extracts of species belonging to other families—*Porites astreoides* (Poritidae), *Pseudodiploria strigosa* (Faviidae), and *Siderastrea siderea* (Rhizangiidae) [46]—which may have some similarity with the SCRiP family. We identified a *SCRiP* in *Dipsastraea lizardensis* (Faviidae), but not in the transcriptome of *P. lutea* and the genomes of *P. australiensis* and *P. rus* (Poritidae) (Supplementary Table S2). However, in sea anemones, *SCRiPs* were detected in three families of the Actinioidea superfamily—Actiniidae, Heteractidae, and Stichodactylidae—with the SCRiP from *M. senile* (*Uniprot*: P0DL60) being unique to the Metridioidea superfamily [28]. In accordance with previous transcriptomic studies [24,36], we did not detect *SCRiPs* outside the Actinioidea superfamily (Edwardsioidea and Metridioidea) (Supplementary Table S2). However, bioinformatic homology searches are dependent on query sequences from public databases, and the sampling of more species outside Actinioidea may potentially lead to the retrieval of novel *SCRiPs*.

Although few genetic data were analyzed from Ctenophora (comb jellies), the absence of *SCRiP* sequences in this phylum may indicate their specificity to venomous organisms [9], i.e., of the phylum Cnidaria. Moreover, metagenomic studies may be interesting to understand if similar *SCRiP* sequences from cnidarian hosts are indeed present in some symbionts, as we detected them in two (12.5%) *Symbiodinium* spp. transcriptomes (Supplementary File S1). For example, a previous study revealed potential phylogenetic evidence for the horizontal gene transfer of a toxin (natterin-4 homologue) from fungi to the coral *A. digitifera* [47]. However, it could be a case of host contamination [48].

### 3.2. The Presence of SCRiPs in Other Cnidarians Should Not Be Discarded

No *SCRiP* sequences were found in the remaining Hexacorallia orders—Corallimorpharia (corallimorpharians), Antipatharia (black corals), and Zoantharia (zoanthids)—or in Ceriantharia (ceriantharians). This is in accordance with a previous transcriptomic analysis of four ceriantharians—although 500 novel toxin-like genes were present, no *SCRiP* sequences were detected [26]. However, the lack of Zoantharia transcriptomes and the low number of assembled genomic data from Ceriantharia and Corallimorpharia might have influenced our results. In addition, Antipatharia data are lacking, and given the similar usage of nematocytes for feeding purposes as stony corals [49], these organisms should be targeted for venom research. Although we only detected *SCRiP*-like sequences in the malacalcyonacean *S. gracillima*, the potential presence of *SCRiPs* in Octocorallia should not be discarded. A previous study assessing nematocyst extracts from three malacalcyonaceans—*Nepthea* sp., *Dendronephthya* sp., and *Heteroxenia fuscescens*—showed low molecular-weight proteins exhibiting several bioactive effects [50].

Moreover, two potential *SCRiPs* were identified in cubozoans (Medusozoa)—*C. sivickisi* and *M. virulenta*—but were not considered for phylogenetic analysis. Contrary to anthozoans, who produce mostly low molecular-weight peptide neurotoxins, medusozoans rely more on enzymatic toxins [15,51].

### 3.3. Proposal of a Novel SCRiP Nomenclature Based on Well-Supported Monophyly

Phylogenetic studies of toxin families can elucidate their evolution and clustering within groups [52,53,54], as observed with other cnidaria toxins [25,27,28]. Given that homologous sequences frequently possess similar functions [27,28,55], this approach is a valuable step for further functional studies. Here, phylogenetic analyses of the SCRiP family revealed four distinct monophyletic gene subfamilies (Figure 3) according to strict discrimination criteria, allowing the proposal of a new nomenclature. This approach managed to group previously identified toxins that were named by their order of discovery, rather than by sequence similarity (Table 2). *SCRiP-α* includes only Acroporidae corals (mainly *Acropora* spp.), and, thus, it may be specific to this family. Contrarily, *SCRiP-β* and *SCRiP-ϒ* were detected in several families of stony corals—Acroporidae, Caryophylliidae, Faviidae, Fungiidae, Merulinidae, Montastraeidae, and Pocilloporidae. *SCRiP-δ*, on the other hand, is divided in two clusters—one formed by sea anemones and another by stony corals of the genus *Acropora* (Figure 4).

These groups can change over time with the discovery of new *SCRiP* sequences, especially *SCRiP-β*, which is the most diverse subfamily and the one with the lowest nucleotide and amino acid percentage identity. Additionally, sea anemones toxins already have a well-defined nomenclature [56], and the continuous detection of peptide toxins from Anthozoa may be a steppingstone for a global nomenclature system.

### 3.4. SCRiPs May Evolve via the “Birth and Death” Gene Evolution

Many toxins evolve according to the “birth and death” gene evolution [4,57] in which a physiological gene duplicates and gains a venomous function, followed by either (i) additional duplications with neo- or sub-functionalization, resulting in large multilocus gene families with distinct roles; or (ii) by pseudogenization, in which some copies lose functionality, suffering the relaxation of selective pressures with a subsequent accumulation of mutations and degeneration [4,58]. Considering the evolution of Hexacorallia [8,11,44,45], the most parsimonious hypothesis suggests that *SCRiP-δ* could be the most basal, given its presence in both Actiniaria and Scleractinia, indicating a potential origin in the common ancestral. Subsequently, *SCRiP-β* and *SCRiP-ϒ* might have emerged in the common ancestral of stony corals via gene duplication and neofunctionalization, followed by the loss of *SCRiP-δ* in the “robust” clade and the birth of *SCRiP-α* in the “complex” clade (Figure 4).

The use of our new retrieved sequences (Supplementary Table S2) as queries for searching future sequenced target genomes and transcriptomes could potentially retrieve additional *SCRiP* sequences, further clarifying the origins of each *SCRiP* subfamily. The absence of *SCRiP* sequences in Octocorallia, Ceriantharia, and the remaining Hexacorallia orders (Zoantharia, Antipatharia, and Corallimorpharia) limits the generalization of our conclusions about gene losses and gains during evolution. Further evidence would be welcome to unequivocally claim that SCRiPs are specific to Actiniaria and Scleractinia.

### 3.5. SCRiPs May Have Different Functions

The ecological role of SCRiPs is currently unknown. Only one study demonstrated that SCRiPs from the stony coral *A. millepora* caused neurotoxicity in zebrafish larvae, but not in blowfly (*Sarcophaga falculata*) larvae [28], suggesting that (i) these toxins may only act on vertebrate receptors for defense of the coral or even for predation of fish larvae; or (ii) blowflies have specific barriers against SCRiPs, and these toxins may target other marine invertebrates, such as zooplanktonic decapods. Stony corals are predated by several species, including corallivorous fish [59,60,61], decapods [59], and crown-of-thorns starfish (COTS) [62]. Interestingly, *Acropora valida* and *Acropora tenuis* were observed to inflict damage through stinging on COTS juveniles, delaying their transition into corallivorous adults [62]. Conversely, stony corals are also effective zooplankton feeders that capture prey or particles via mucus adhesion or tentacle grabbing through nematocyst discharge [63,64]. In conclusion, stony corals may use their toxins, including SCRiPs, for deterrence of corallivorous species or predation. Curiously, *Acropora* spp. contain SCRiPs from three subfamilies—*SCRiP-α*, *SCRiP-ϒ*, and *SCRiP-δ*—that are present in distinct life stages (Figure 4 and Supplementary Table S2) and, thus, may have specific functions. Toxins from the same family can have distinct functions within different life stages in cnidarians, which correlates with transitions from defensive to predatory phases or with changes in feeding ecology [65,66,67,68]. In our study, *SCRiPs* were also detected in gastrula, larvae, and adult stages from other stony corals (Figure 4).

In sea anemones, the ecological function of toxins can be slightly inferred by their anatomical localization. Enzymatic toxins are highly expressed in the gastrodermis and mesenteric filaments for digestive roles, while neurotoxins and membrane-active toxins are more expressed in the epidermis and tentacles, supporting prey immobilization and predator deterrence [2,36,69,70]. Here, *SCRiP-δ* was the only subfamily detected in sea anemones, being mainly retrieved from the transcriptomic data of adults’ tentacles (Figure 4). Hence, *SCRiP-δ* may have an important role in predation or defense in these organisms. In a previous study, *SCRiPs* from sea anemones were found to be massively upregulated in the acrorhagi [36]. Although we found possible *SCRiP*-like sequences in the acrorhagi of *A. elegantissima*, they did not pass our filtering criteria (Supplementary Table S2). Future studies should test whether *SCRiPs* are expressed in both structures, as they can also have a role in intraspecific competition [36]. Also, retrieving juvenile samples from sea anemones may confirm if SCRiPs are only produced by adult individuals. In the ML gene tree, all sea anemone sequences clustered together, forming a sister group with the *Acropora* sp. cluster within the *SCRiP-δ* subfamily (Figure 3). Given that Amil_SCRiP2 (Uniprot: C0H691) belongs within this clade (Table 2) and had strong neurotoxic effects on zebra fish larvae [28], SCRiPs from sea anemone may be likewise potent and have comparable functions.

### 3.6. SCRiP Subfamilies Evolve under Distinct Selective Pressures, Suggesting Potential Neofunctionalizations

In contrast with evolutionary younger lineages, such as snakes and cone snails, whose venom evolution is driven by the strong influence of diversifying positive selection, purifying selection is broadly observed within toxin families of ancient diverging venomous animals, such as cnidarians and coleoids [71,72], in which toxin function and potency is well preserved after an initial period of expansion. However, when venomous animals venture into novel ecological niches and encounter new types of prey and predators, new selective pressures act on venom proteins to efficiently target them [71]. Some episodically adaptive sites may be reflective of such shifts in ecology [73], and some clades within the same toxin family can evolve under the influence of diversifying positive selection [52], as seen in sea anemone neurotoxins [28] and jellyfish toxins [25]. Curiously, Jouiaei, Sunagar, et al. (2015) examined the impact of selection on seven SCRiP sequences (five from stony corals and two from sea anemones), which were under the influence of purifying selection. However, seven sites across these sequences displayed signs of episodic diversifying positive selection [28].

In contrast, we assessed the influence of selection for each *SCRiP* subfamily individually. All *SCRiP* subfamilies were generally under purifying selection, except for *SCRiP-α*, which is under diversifying positive selection. However, all *SCRiP* subfamilies show signals of similar PSSs across tests (Table 3 and Supplementary Table S3). Currently, there is no available information regarding functionally important residues in SCRiP sequences, thus hindering the interpretation of the evolutionary importance of these PSS. The manipulation of specific SCRiP residues, complemented with functional testing, will elucidate which residues are more important for bioactivity.

The higher prevalence of purifying selection on *SCRiP-δ* implies its environmentally functional relevancy, which is in accordance with their basal origin, as supported by the most parsimonious hypothesis (Figure 4). This may be explained by the higher potency of Amil_SCRiP2 (Uniprot: COH691) [28] within this group. In contrast, signals of diversifying positive selection across sites in *SCRiP-α* might indicate an ongoing adaptation, as Amil_SCRiP3 (Uniprot: COH692), which belongs within this group, showed lower potency [28]. Future studies should address how environmental factors and SCRiP toxin targets are influencing selection. Also, distinct populations can modulate their venom production levels [74] via the action of distinct selective pressures, leading to the decrease in the expression or even loss of toxin genes at the genome level.

## 4. Conclusions

Here, we undertook the first analysis of the evolutionary history of SCRiPs, and, to our knowledge, the first attempt to explore the evolution of a specific toxin family derived from stony corals. Through an extensive taxon sampling and a strict bioinformatic approach, we annotated 168 novel putative *SCRiP* genes from 36 stony corals (Scleractinia) and 12 sea anemones (Actiniaria). Our findings allowed for the proposal of a new SCRiP nomenclature comprising four distinct subfamilies, with one of them demonstrating influence from diversifying positive selection. Our results also led to a wider understanding of the distribution and evolution of SCRiPs within Anthozoa. Although a broader range of scleractinian-like toxins were assessed due to the available genomic and transcriptomic data, the stringent parameters employed in our bioinformatic pipeline provide confidence in the fidelity of the suggested subfamily structure. The sampling of more species, coupled with highly accurate sequencing technologies and the improvement of genome and transcriptome assembly, will further support the identification of additional SCRiPs candidates. We expect that our study will assist future proteomic and functional research on this enigmatic neurotoxin family from stony corals and sea anemones.

## 5. Materials and Methods

### 5.1. Species Assortment and Sequence Gathering

A total of 119 genomes (Scleractinia (*n* = 36), Actiniaria (*n* = 13), Zoantharia (*n* = 29), Corallimorpharia (*n* = 1), Octocorallia (*n* = 10), and Medusozoa (*n* = 30)); and 104 transcriptomes (Scleractinia (*n* = 37), Actiniaria (*n* = 19), Corallimorpharia (*n* = 3), Ceriantharia (*n* = 1), Octocorallia (*n* = 10), and Medusozoa (*n* = 30)) were extracted from NCBI and GitHub publicly available databases: https://www.ncbi.nlm.nih.gov/assembly/ (accessed on 9 January 2023) for genomes; and https://www.ncbi.nlm.nih.gov/nuccore/ (accessed on 14 February 2023) and https://github.com/XylotrupesGideon/Cnidarian-Sequence-resources (accessed on 14 February 2023) [75] for transcriptomes. Also, 6 genomes and 4 transcriptomes of the comb jellies Ctenophora (non-venomous) and 7 genomes and 16 transcriptomes of the algae *Symbiodinium* sp. (cnidaria symbionts) were retrieved from the same databases (Supplementary Table S1). SCRiP protein sequences were searched on the VenomZone database: https://venomzone.expasy.org (accessed on 20 February 2023) and retrieved from UniProt (*n* = 15) [76], as well as predicted genomic sequences from NCBI (*n* = 11). Only SCRiPs with a well-defined eight-cysteine framework were used for further analyses (*n* = 24) (Table 1), excluding SCRiP-like sequences, such as Ueq-12 (UniProt: C0HK26). Previously described SCRiPs from *H. actiniformis* (Hact_SCRiP1; UniProt: 7LX4) [41] and *A. viridis* (Avir_SCRiP; UniProt: PODL61) [28] were not considered due to the lack of a nucleotide sequence and higher divergency, respectively (Supplementary Table S2).

### 5.2. Ortholog Identification

The protein2genome option of the Exonerate v2.4.0 [77] software was used to identify putative orthologous *SCRiPs* from the genomes. Both complete coding sequences (CDSs) and partial sequences with high similarity with the queries (similar intron size and cysteine framework) were extracted. TransDecoder v5.5.0 [78] software was used to predict long open reading frames (ORFs) from the transcriptomes to create a searchable putative peptide database, with a minimum sequence length of 60 amino acids (parameter-m set to 60). A hidden Markov model (HMM) (Supplementary File S3) was established with the SCRiP queries, using HHMER v3.3.2 [79] to extract CDSs from the transcriptomes, with an e-value cutoff of 1 × 10^−5^. HMM-based methods are more accurate for homology search, as they provide important information about protein conserved regions [55]. All CDSs and partial sequences with a maximum length of 100 amino acids were considered for downstream analyses (Supplementary Table S2).

### 5.3. Sequence Filtering and Gene Validation

The CD-HIT v4.8.1 [80] software was used to identify equal protein sequences (parameter-c set to 1) in order to remove redundant sequences. We then used *SignalP* v6.0 [81] to predict the presence of a signal peptide (>65% probability), as toxins are secretory (Supplementary Table S2); and DeepTMHMM v1.0.24 [82] confirmed if these sequences had 0 or 1 transmembrane domains (TDs), with the only known TD of SCRiPs corresponding to the signal peptide (Supplementary File S4).

A final BLASTp search using NCBI-BLAST+ v2.12 [83] was conducted against a customized database of the ToxProt Animal Toxin Annotation from UniProt/SwissProt [76,84] and the NCBI protein database: https://www.ncbi.nlm.nih.gov/protein (accessed on 30 March 2023), using the keywords “Cnidaria” AND (“Toxin” OR “Venom”) with an e-value cutoff of 1 × 10^−5^ to determine if the best match corresponds to previously described SCRiPs. Those with less than 40% percentage identity were removed, except for putative *SCRiP*-like sequences from the octocoral *Scleronephthya gracillima* (Malacalcyonacea) (32.50% to 37.98% percentage identity). Only Anthozoan sequences were considered. Sequences that met these criteria were considered for phylogenetic and selection analyses (Supplementary Table S2).

### 5.4. Phylogenetic Analysis and SCRiP Family Classification

A codon multiple sequence alignment (MSA) of the putative sequences, queries, and outgroups (*n* = 192) was created, employing the MAFFT algorithm and 100 bootstrap repeats on the GUIDANCE2 webserver [85]. The alignment was refined by removing codon positions with less than 10% of informative codons (non-ambiguous codons or gaps) (Supplementary File S5). The MSA was submitted to a nucleotide substitution model analysis in the JModelTest v2.1.7 software [86]. The likelihood of different nucleotide substitution models was calculated, and the best-fit model was chosen after the corrected Akaike information criterion (AICc) [87] (Supplementary File S6). Phylogenetic inference was performed by employing the TVM+I+G model, using the maximum-likelihood (ML) algorithm in the IQ-TREE v2.0.7 [88] software, applying 10,000 replicates for ultrafast bootstraps [89], 10,000 replicates for parametric approximate likelihood-ratio test (SH-aLRT) [90], and an approximate Bayes test [91] (Figure 3 and Supplementary Figure S1). Putative *SCRiP*-like sequences from the octocoral *S. gracillima* were used as an outgroup, given their basal separation from Hexacorallia within Anthozoa (Figure 1).

*SCRiP* subfamilies were defined according to well-established monophyly, high bootstrap support values in the divergence nodes of the phylogeny (bs > 95%) and nucleotide identity (ni) higher and less than 55% within and between groups, respectively. Protein identity (pi) was also calculated. Both percentage identities were calculated using the Geneiou*s* v11.1.5 software [92].

### 5.5. Selection Analysis

Prior to the selection analysis, the predicted signal peptide was removed from *SCRiP* sequences, as this region is likely evolving under different selective pressures than the mature toxin sequence (Supplementary Table S2) [72]. Given that larger datasets with highly similar sequences may compromise results [93], the total number of candidate genes was reduced by randomly removing sequence paralogs from each species in triplicate, retaining the species diversity. Also, the sequences from *M. senile* (UniProt: P0DL60) and *Favites colemani* (GIVN01215367.p1) were removed due to high divergency from their orthologs.

An MSA was created for each *SCRiP* subfamily, using the methodology mentioned above, removing codon positions with less than 50% of informative codons (Supplementary File S2). Selection analyses were conducted separately in each replicate of each *SCRiP* subfamily, using the Hyphy v2.5 package [94] on the DataMonkey web server [95] (Table 3 and Supplementary Table S3). MEME [73] was used to determine sites under episodic positive selection, using a mixed-effects ML approach. To further infer non-synonymous (*dN*) and synonymous (*dS*) substitution rates on a per-site basis, three additional methods were used: FEL [96]—ML approach; FUBAR [97]—Bayesian approach; and SLAC [96]—combination of ML and counting approaches. Positive selection predictions were accepted if the posterior probability was PP > 0.95 in FUBAR and the *p*-value was *p* < 0.05 in the remaining tests. The overall *dN*/*dS* (*ω*) ratio values for each clade were estimated using the global fitted MG94xREV model [98] included in the SLAC analysis.

## Figures and Tables

**Table 1:** Cnidaria SCRiPs (*n* = 24) retrieved from publicly available databases used as queries for an ortholog search and phylogenetic analysis. Toxin names of predicted genomic sequences retrieved from NCBI were conferred according to species name and similarity to reviewed SCRiPs.

Toxin Name	Species (Order)	Protein Entry (Length aa)	Cysteine Framework	Reference
Amil_SCRiP1 *	*A. millepora* (S)	C0H690 (89)	C_(6)_C_(6)_C_(7)_C_(5)_C_(6)_CCC_(12)_	[33]
Amil_SCRiP2 *	*A. millepora* (S)	C0H691 (79)	C_(6)_C_(6)_CP_(5)_C_(6)_C_(6)_CCC_(2)_	[33]
Amil_SCRiP3 *	*A. millepora* (S)	C0H692 (83)	C_(6)_C_(6)_CP_(5)_C_(5)_C_(6)_CCC_(2)_	[33]
Ofav_SCRiP1 *	*O. faveolata* (S)	C1KIY9 (79)	C_(6)_C_(4)_CP_(5)_C_(6)_C_(6)_CCC_(2)_	[33]
Ofav_SCRiP2 *	*O. faveolata* (S)	C1KIZ0 (68)	C_(6)_C_(6)_CP_(6)_C_(5)_C_(6)_CCC_(2)_	[33]
Ofav_SCRiP4 *	*O. faveolata* (S)	C1KIZ3 (81)	C_(7)_C_(5)_CP_(5)_C_(4)_C_(6)_CCC_(4)_	[33]
Ofav_SCRiP5 *	*O. faveolata* (S)	C1KIZ4 (68)	C_(6)_C_(6)_CP_(6)_C_(5)_C_(6)_CCC_(2)_	[33]
Ofav_SCRiP6 *	*O. faveolata* (S)	C1KIZ5 (81)	C_(7)_C_(6)_CP_(5)_C_(5)_C_(5)_CCC_(5)_	[33]
Ofav_SCRiP8 *	*O. faveolata* (S)	B2ZG38 (74)	C_(6)_C_(6)_CP_(3)_C_(4)_C_(6)_CCC_(2)_	[33]
Mcap_SCRiP1a *	*M. capitata* (S)	C0H693 (81)	C_(6)_C_(6)_CP_(5)_C_(4)_C_(5)_CCC_(2)_	[33]
Mcap_SCRiP1b *	*M. capitata* (S)	C0H694 (81)	C_(6)_C_(6)_CP_(5)_C_(4)_C_(5)_CCC_(2)_	[33]
Msen_SCRiP *	*M. senile* (A)	P0DL60 (69)	C_(6)_C_(6)_CP_(5)_C_(5)_C_(7)_CCC_(3)_	[28]
Aten_SCRiP	*A. tenebrosa* (A)	A0A3P8MJV5 (98)	C_(6)_C_(6)_C_(6)_C_(5)_C_(6)_CCC_(7)_	[36]
Amil_SCRiP3like	*A. millepora* (S)	XP_044181782.1 (84)	C_(6)_C_(6)_C_(6)_C_(5)_C_(5)_CCC_(4)_	Predicted
Amil_SCRiP1like	*A. millepora* (S)	XP_044164975.1 (89)	C_(6)_C_(6)_C_(7)_C_(5)_C_(6)_CCC_(12)_	Predicted
Spis_SCRiP1like	*S. pistillata* (S)	XP_022810608.1 (89)	C_(6)_C_(6)_C_(7)_C_(5)_C_(6)_CCC_(12)_	Predicted
Ofav_SCRiP8like2	*O. faveolata* (S)	XP_020613277.1 (87)	C_(6)_C_(6)_CP_(3)_C_(4)_C_(6)_CCC_(6)_	Predicted
Ofav_SCRiP6like2	*O. faveolata* (S)	XP_020610104.1 (80)	C_(8)_C_(6)_CP_(5)_C_(3)_C_(5)_CCC_(6)_	Predicted
Adig_SCRiP3like1	*A. digitifera* (S)	XP_015749964.1 (84)	C_(6)_C_(6)_C_(6)_C_(5)_C_(5)_CCC_(4)_	Predicted
Adig_SCRiP3like2.1	*A. digitifera* (S)	XP_015749963.1 (84)	C_(6)_C_(6)_C_(6)_C_(5)_C_(5)_CCC_(4)_	Predicted
Adig_SCRiP3like2.2	*A. digitifera* (S)	XP_015780210.1 (84)	C_(6)_C_(6)_C_(6)_C_(5)_C_(5)_CCC_(4)_	Predicted
Adig_SCRiP1	*A. digitifera* (S)	XP_015773994.1 (89)	C_(6)_C_(6)_C_(7)_C_(5)_C_(6)_CCC_(12)_	Predicted
Adig_SCRiP3	*A. digitifera* (S)	XP_015772574.1 (83)	C_(6)_C_(6)_CP_(5)_C_(5)_C_(6)_CCC_(2)_	Predicted
Adig_SCRiP3like3	*A. digitifera* (S)	XP_015765712.1 (89)	C_(6)_C_(6)_C_(6)_C_(4)_C_(5)_CCC_(10)_	Predicted

* Reviewed SCRiPs retrieved from UniProt. Amil—Acropora millepora. Adig—Acropora digitifera. Aten—Actinia tenebrosa. Mcap—Montipora capitata. Msen—Metridium senile. Ofav—Orbicella faveolata. Spis—Stylophora pistillata. S—Scleractinia. A—Actiniaria. (n)—n represents the number of amino acids (aa) between cysteines © in each SCRiP sequence. P—proline.

**Table 2:** Placement of previously described SCRiP sequences in each *SCRiP* subfamily, together with information about the percentage of *SCRiPs* found in each stony coral (Scleractinia) and sea anemone (Actiniaria) families (Hexacorallia).

*SCRiP* Subfamily	Previous SCRiP Sequences from Uniprot (Entry)	Previous SCRiP Sequences from NCBI (Entry)	Hexacorallia Families (Percentage of *SCRiP* Sequences)
*SCRiP-α*	Amil_SCRiP3 (C0H692) Mcap_SCRiP1a (C0H693) Mcap_SCRiP1b (C0H694)	Adig_SCRiP3 (XP_015772574.1) Amil_SCRiP3like (XP_044181782.1) Adig_SCRiP3like1 (XP_015749964.1) Adig_SCRiP3like2.1 (XP_015749963.1) Adig_SCRiP3like2.2 (XP_015780210.1) Adig_SCRiP3like3 (XP_015765712.1)	Acroporidae (100%)
*SCRiP-β*	Ofav_SCRiP1 (C1KIY9) Ofav_SCRiP4 (C1KIZ3) Ofav_SCRiP6 (C1KIZ5) Ofav_SCRiP8 (B2ZG38)	Ofav_SCRiP6like2 (XP_020610104.1) Ofav_SCRiP8like2 (XP_020613277.1)	Acroporidae (2.22%) Caryophylliidae (22.22%) Faviidae (2.22%) Fungiidae (2.22%) Merulinidae (51.11%) Montastraeidae (8.89%) Pocilloporidae (11.11%)
*SCRiP-ϒ*	Amil_SCRiP1 (C0H690) Ofav_SCRiP2 (C1KIZ0) Ofav_SCRiP5 (C1KIZ4)	Amil_SCRiP1like (XP_044164975.1) Spis_SCRiP1like (XP_022810608) Adig_SCRiP1 (XP_015773994.1)	Acroporidae (50.00%) Caryophylliidae (18.75%) Merulinidae (25.00%) Pocilloporidae (6.25%)
*SCRiP-δ*	Amil_SCRiP2 (C0H692) Msen_SCRiP (P0DL60) *	Aten_SCRiP (ATY39986.1) *	Acroporidae (38.70%) Actiniidae * (19.35%) Heteractidae * (12.90%) Stichodactylidae * (25.81%) Metridiidae * (3.23%)

Uniprot and NCBI entries are represented in brackets. * Sea anemones (Actiniaria) families.

**Table 3:** Site-models selection analyses of each *SCRiP* subfamily. Each group was randomly reduced to three replicates, maintaining the species diversity (see Supplementary File S2). Global ω was calculated using the MG94xREV model included in the SLAC analysis.

*SCRiP* Subfamily (*n*)	MEME (*p* < 0.05)	FEL (PP > 95%)	FUBAR (*p* < 0.05)	SLAC (*p* < 0.05)	Global ω (MG94xREV)
*SCRiP-α* (19)	8	ω > 1:2; ω < 1:2	ω > 1:3; ω < 1:0	ω > 1:0; ω < 1:0	1.36
	8	ω > 1:3; ω < 1:5	ω > 1:5; ω < 1:1	ω > 1:0; ω < 1:2	1.28
	4	ω > 1:3; ω < 1:5	ω > 1:6; ω < 1:2	ω > 1:2; ω < 1:3	1.26
*SCRiP-β* (15)	4	ω > 1:0; ω < 1:16	ω > 1:2; ω < 1:12	ω > 1:3; ω < 1:13	0.647
	5	ω > 1:0; ω < 1:14	ω > 1:1; ω < 1:12	ω > 1:0; ω < 1:9	0.595
	7	ω > 1:2; ω < 1:12	ω > 1:2; ω < 1:11	ω > 1:0; ω < 1:10	0.673
*SCRiP-ϒ* (18)	3	ω > 1:2; ω < 1:10	ω > 1:5; ω < 1:6	ω > 1:0; ω < 1:3	0.688
	2	ω > 1:1; ω < 1:12	ω > 1:2; ω < 1:5	ω > 1:0; ω < 1:6	0.599
	4	ω > 1:2; ω < 1:14	ω > 1:3; ω < 1:6	ω > 1:0; ω < 1:6	0.569
*SCRiP-δ* (22)	4	ω > 1:1; ω < 1:8	ω > 1:2; ω < 1:4	ω > 1:0; ω < 1:4	0.605
	3	ω > 1:2; ω < 1:10	ω > 1:1; ω < 1:5	ω > 1:0; ω < 1:5	0.577
	3	ω > 1:2; ω < 1:11	ω > 1:1; ω < 1:5	ω > 1:0; ω < 1:5	0.545

*n*—number of SCRiPs; *p*—*p*-value; PP—posterior probability.

## Data Availability

The data used in this study was not de novo generated, but extracted from publicly available data (genomes and transcriptomes). In any case, the extracted sequences are available in the Supplementary Table S2 (both protein and nucleotide sequences, with and wihtout signal peptides), in the sections “Final Database Transcriptomes” and “Final Database Genomes”.

## Supplementary Materials

The following supporting information can be downloaded at https://www.mdpi.com/article/10.3390/toxins16020075/s1, Figure S1: Maximum Likelihood (ML) gene tree of *SCRiP* nucleotide sequences with statistics for the ultrafast bootstraps, approximate likelihood-ratio test (SH-aLRT), and approximate Bayes Test; Table S1: Transcriptomic and genomic data from Cnidaria, Ctenophora, and *Symbiodinium* sp.; Table S2: Data from ortholog gene search, filtering, and validation; Table S3: Selection analysis data with retrieved PSS; File S1: BLASTp output of *SCRiPs* retrieved from *Symbiodinium* sp. transcriptomes; File S2: Triplicate MSA of each *SCRiP* subfamily submitted to selection analysis; File S3: Hidden Markov model (HMM) of SCRiPs retrieved from pubic databases (Uniprot and NCBI); File S4: DeepTMHMM output of filtered *SCRiP* sequences; File S5: Refined MSA of the ortholog *SCRiPs* (*n* = 192) submitted to phylogenetic analysis; File S6: JModelTest output with the best-fit models after the Akaike information criterion (AICc).
