# Supplementary material for: Evolutionary Analysis of Cnidaria Small Cysteine-Rich Proteins (SCRiPs), an Enigmatic Neurotoxin Family from Stony Corals and Sea Anemones (Anthozoa: Hexacorallia)
Source: Toxins (Basel). 2024 Feb 2;16(2):75. doi: 10.3390/toxins16020075 (PMC10892658; doi:10.3390/toxins16020075)
Supplement: Supplementary file 1 [file toxins-16-00075-s001.zip › Figure_S1.pdf]

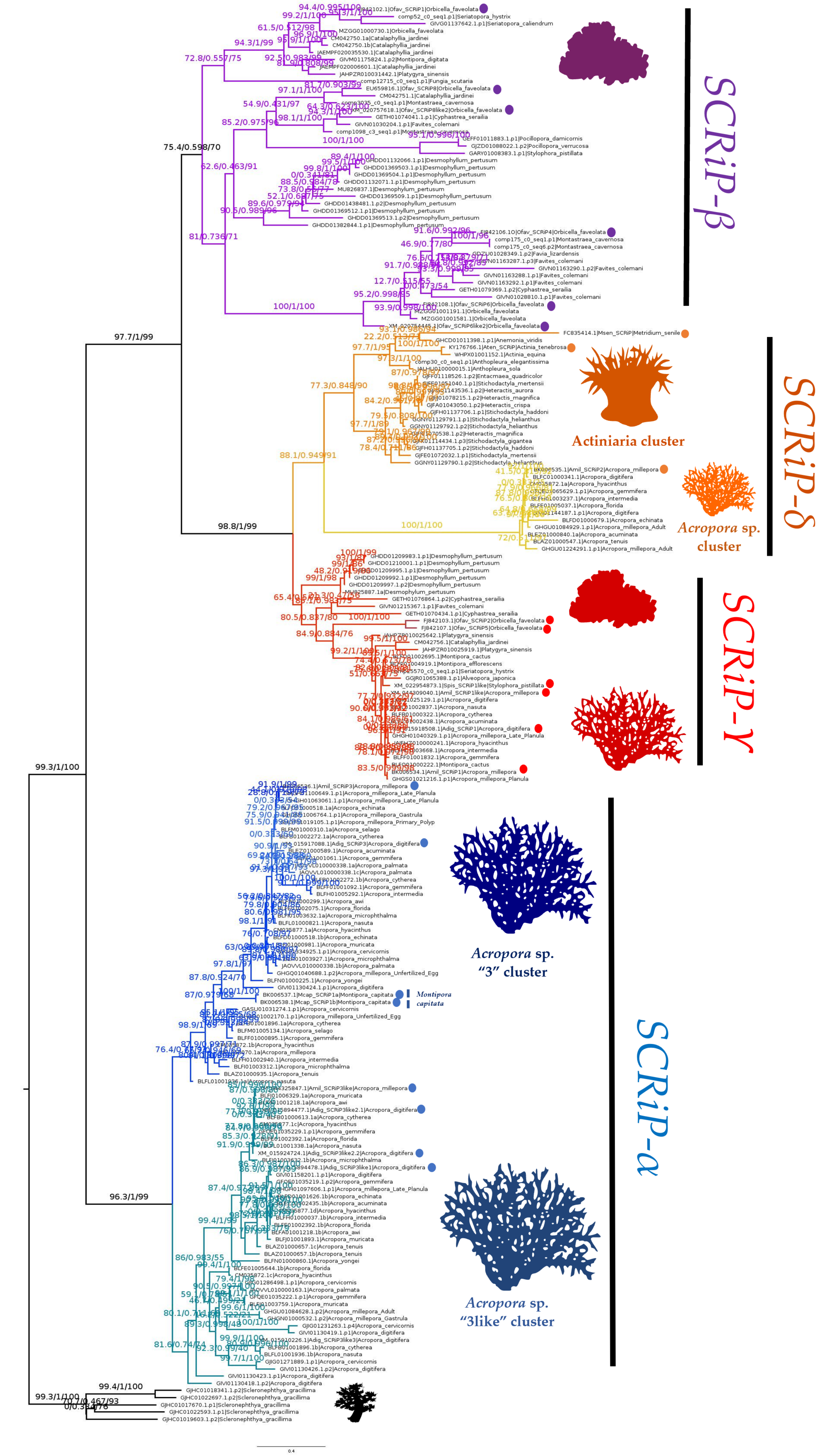

Figure S1. Maximum Likelihood (ML) gene tree of *SCRiP* nucleotide sequences. The phylogenetic tree was constructed using IQ-Tree. Statistics for the parametric approximate likelihood-ratio test (SH-aLRT) (10,000 replicates), approximate Bayes test and ultrafast bootstraps (10,000 replicates) were included in the divergence nodes, respectively. Colored dots mark SCRiP sequences previously identified in other studies and available in public databases (Uniprot and NCBI).
